# Characterization and differential expression of microRNAs in the ovaries of pregnant and non-pregnant goats (*Capra hircus*)

**DOI:** 10.1186/1471-2164-14-157

**Published:** 2013-03-07

**Authors:** Xiao-Dong Zhang, Yun-Hai Zhang, Ying-Hui Ling, Ya Liu, Hong-Guo Cao, Zong-Jun Yin, Jian-Ping Ding, Xiao-Rong Zhang

**Affiliations:** 1 Anhui Provincial Laboratory of Local Animal Genetic Resources Conservation and Biobreeding, No. 130 Changjiang west road, Hefei 230036, P. R. China; 2 College of Animal Science and Technology, Anhui Agricultural University, No. 130 Changjiang west road, Hefei 230036, P. R. China

**Keywords:** MicroRNA, Solexa sequencing, Ovary, Anhui white goat

## Abstract

**Background:**

Ovarian follicular development and hormone secretion are complex and coordinated biological processes which will usually be altered during pregnancy. Ovarian function is tightly regulated by a multitude of genes, and also by some specific miRNAs. It is necessary to identify the differentially expressed miRNAs in the ovaries of pregnant and non-pregnant mammals, in order to further understand the role of miRNA-mediated post-transcriptional regulation in mammalian reproduction. Here, we performed a comprehensive search for hircine miRNAs using two small RNA sequencing libraries prepared from the ovaries of pregnant and non-pregnant goats.

**Results:**

617 conserved and 7 putative novel miRNAs were identified in the hircine ovaries. A total of 471 conserved miRNAs (76.34%) were co-expressed in both pregnant and non-pregnant libraries, and 90 pregnancy-specific and 56 non-pregnancy-specific conserved miRNAs were identified. Additionally, 407 unique miRNAs (65.96%) were significantly differentially expressed in the pregnant and non-pregnant libraries, of which 294 were upregulated and 113 were downregulated in the pregnant library compared to the non-pregnant library. Further analysis showed that miR-143 was predicted to bind to the target sequences of Frizzled-6 and -3 receptor genes in the Wnt/beta-catenin signaling pathway, and let-7b may target the Activin receptor I and Smad 2/3 genes in the TGF-beta signaling pathway. The expression level of 5 randomly selected miRNAs were analyzed by quantitative real-time PCR (q-PCR), and the results demonstrated that the expression patterns were consistent with the Solexa sequencing results.

**Conclusions:**

The identification and characterization of differentially expressed miRNAs in the ovaries of pregnant and non-pregnant goats provides important information on the role of miRNA in the regulation of the ovarian development and function. This data will be helpful to facilitate studies on the regulation of miRNAs during mammalian reproduction.

## Background

MicroRNAs (miRNAs) are a group of single-stranded noncoding 21–24 nt RNAs which are involved in diverse aspects of eukaryotic biology including reproduction, development, pathogenesis, cell proliferation, apoptosis and lipometabolism by pairing to mRNAs which mainly results in target-specific post-transcriptional repression [1-4]. Recent research has demonstrated that miRNAs are involved in the regulation of mammalian reproduction, especially the regulation of ovarian function [4,5]. The Dicer1-deficient mice which lost ~75% of Dicer1 mRNA expression demonstrated female infertility, and were predicted to reduce angiogenesis in the corpus luteum because of the loss of miR-17-5p and let-7b which regulate the expression of tissue inhibitor of metalloproteinase 1 [6,7]. When the mouse granulosa cells were treated with the human chorionic gonadotropin (hCG), the miR-132 and miR-212 expression were upregulated, and knockdown of these miRNAs increased the expression of C-terminal binding protein 1 (CtBP1) in granulosa cells [8]. CtBP1 was recently shown to regulate adrenal steroidogenesis, in conjunction with steroidogenic factor-1 [9]. Additionally, massive parallel sequencing in the newborn mouse ovaries showed the expression of 398 known miRNAs, among which the X-linked mir-503, mir-672 and mir-465 family were found to be preferentially expressed in the testes and ovaries which play important roles in folliculogenesis and spermatogenesis, respectively [10].

Pregnancy is a complex reproductive process, which is tightly regulated by various endocrine factors and a large number of genes. The ovaries play an important role during pregnancy. There are significant differences in the activity and endocrine characteristics of the ovary during pregnancy and non-pregnancy [11-13]. In the non-pregnant phase, ovulation is normal and estrogen secretion dominates; whereas ovulation is temporarily suspended during pregnancy and progesterone secretion gradually increases to maintain pregnancy. In the present study, we characterized and investigated the differential expression of miRNAs in the ovaries of pregnant and non-pregnant goats using deep sequencing technology. The result will help to further understand the role of miRNAs in reproductive biological processes, including follicular development, hormone secretion, luteinization and the maintenance of pregnancy, and also may help to identify miRNAs which could be potentially used to regulate hircine reproduction and breeding practice in the future.

## Results

### Overview of sequencing data

**Figure 1:**
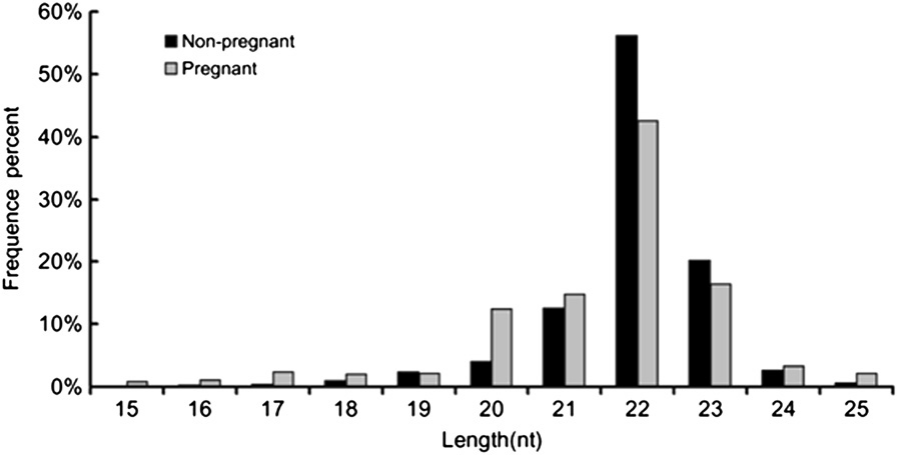
Frequency distribution of sequence lengths of the Solexa sequencing results.

**Figure 2:**
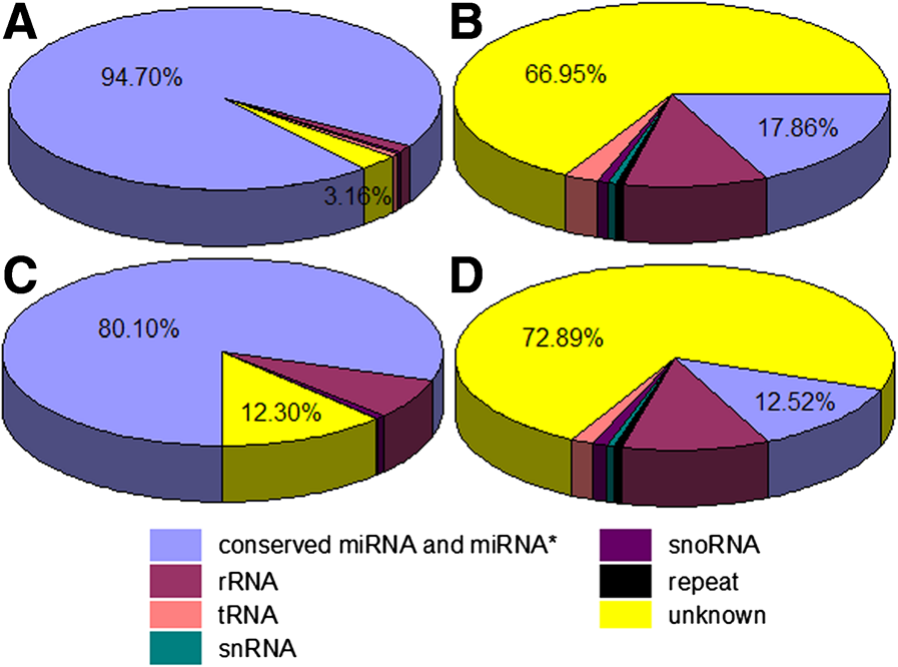
**Composition of small RNA classes of the Solexa sequencing results.** (**A**) Total number of reads in the non-pregnant library. (**B**) Total number of unique sequences in the non-pregnant library. (**C**) Total number of reads in the pregnant library. (**D**) Total number of unique sequences in the pregnant library.

In order to identify differentially expressed miRNAs in the ovaries of pregnant and non-pregnant goats, two small RNA libraries were constructed by Solexa sequencing. The results of 9.98 million (M) and 11.21 M total reads were obtained from the ovarian libraries of pregnant and non-pregnant goats, respectively. After removing the low quality and adaptor sequences, a total of 9.23 M and 11.01 M clean reads were ultimately obtained. Subsequently, all identical sequence reads were classified as groups, and we obtained 0.38 M and 0.20 M unique sequences associated with individual sequence reads. The size distribution of the reads was similar between the two libraries (Figure 1). The majority of the small RNAs were 21–24 nt in size. Sequences 22 nt in length, the typical size of Dicer-derived products [14], accounted for 42.54% and 55.69% of the total sequence reads in the pregnant and non-pregnant ovarian libraries. The composition of the RNA classes in each library is shown in Figure 2 and Additional file 1. Conserved miRNAs accounted for 80.10% and 94.70% of the total sequence reads (Figure 2A and C), and 12.52% and 17.86% of the unique sequence reads (Figure 2B and D) in the pregnant and non-pregnant small RNA libraries, respectively. The rRNA, tRNA, snRNA, snoRNA and repeat DNA total accounted for 7.61% and 2.14% of the total sequence reads, and 5.60% and 15.18% of the unique sequence reads in the pregnant and non-pregnant libraries, respectively (Additional file 1). A total of 561 and 527 conserved miRNAs and miRNA*s (originating from the hairpin pre-miRNA arm opposite to the annotated miRNA containing arm) were identified in the pregnant and non-pregnant libraries, respectively (Additional file 2 and Additional file 3). After grouping the identical sequences, a total of 617 unique miRNAs and miRNA*s were obtained from both libraries. The highest fraction of unique reads (72.89% and 66.95% in the pregnant and non-pregnant libraries) was attributed to unannotated sequences (Figure 2B and D). These sequences were not mapped to any known reference databases. Similar to those of miRNAs, the major unannotated sequences were 20–22 nt in size. However, a considerable part of the unannotated sequences were concentrated in 15–19 and 23–25 nt in size (Additional file 4: Figure S1), indicating a potential role of these small RNAs in hircine ovaries. These results indicated that the small RNA libraries were highly enriched in miRNA sequences, and that there was also a less abundant, but much more diverse, class of small RNAs which may represent other classes of small non-coding RNAs (ncRNAs), consistent with the previous studies [14,15].

### Conserved microRNAs and microRNA*s

To identify conserved miRNAs in hircine ovaries, the dataset was compared to the known mammalian miRNAs (miRNA precursors and mature miRNAs) in miRBase 18.0 (http://www.mirbase.org). A total of 36501 and 47316 unique sequences in the non-pregnant and pregnant libraries were mapped to known mammalian miRNAs in miRBase 18.0, respectively. Considering one or two mismatches between sequences, 508 and 535 conserved miRNAs were identified in the non-pregnant and pregnant libraries, respectively.

Furthermore, 19 miRNA*s were obtained in the non-pregnant library, of which 18 duplex-like miRNA: miRNA* pairs were identified, and 26 miRNA*s in the pregnant library, of which 22 duplex-like miRNA: miRNA* pairs were identified. The expression levels of the majority miRNA*s were lower than the corresponding miRNAs. For example, the read counts of miR-21* in the non-pregnant and pregnant libraries were 81 and 179, compared to the read counts of 43703 and 138637 for miR-21, respectively. However, the expression levels of some miRNA*s were significantly higher than the corresponding miRNAs, such as miR-199b*, suggesting that miR-199b* functions during hircine ovarian development [16]. Some miRNA*s and the corresponding miRNAs were generated at similar levels in both libraries, such as miR-1343, miR-142, miR-9 and miR-2411, (Table 1).

**Table 1:** Comparison of the read counts for miRNAs and the corresponding miRNA*s

**miRNA ID**	**miRNA**		miRNA*	
	**Pregnant library**	**Non-pregnant library**	**Pregnant library**	**Non-pregnant library**
miR-99a	745329	30283	1498	1041
let-7a	247959	2356002	47	51
miR-21	138637	43703	179	81
miR-24	107152	10301	123	23
miR-140	94305	203114	81169	144750
miR-27b	51810	8668	345	95
miR-151	30704	1727	51626	1011
miR-126	21371	164	3978	797
miR-424	20055	3031	484	1131
miR-455	7780	1989	6355	1146
miR-374a	1161	902	224	155
miR-199b	439	1224	39305	194315
miR-2483	316	21	278	9
miR-1343	303	0	362	0
miR-142	200	975	34	2
miR-9	164	62	12	12
miR-2411	125	29	69	2
miR-1434	101	0	126	0
miR-2330	38	0	1	0
miR-2331	6	9	13	3
miR-545	6	3	44	9
miR-219	6	0	3	0
miR-199a	0	0	13887	148870
miR-2370	0	0	2	0
miR-2454	0	0	12	0
miR-376a	0	0	66	0

### Identification of putative miRNAs

Though the substantial goat genome sequence data is currently short, it is feasible to identify putative miRNAs by means of alignment with goat expressed sequence tags (ESTs). Two goat putative miRNAs with a total of 24 read counts, and five goat putative miRNAs with a total of 43 read counts were predicted in pregnant and non-pregnant libraries. The RNA stem loop structures and the positions of the reads were shown in Additional file 5 and Additional file 4: Figure S2. These seven putative miRNAs were not analyzed further, as their expression levels were too low in the two small RNA libraries.

### Differential expression of miRNAs in the ovaries of pregnant and non-pregnant goats

**Figure 3:**
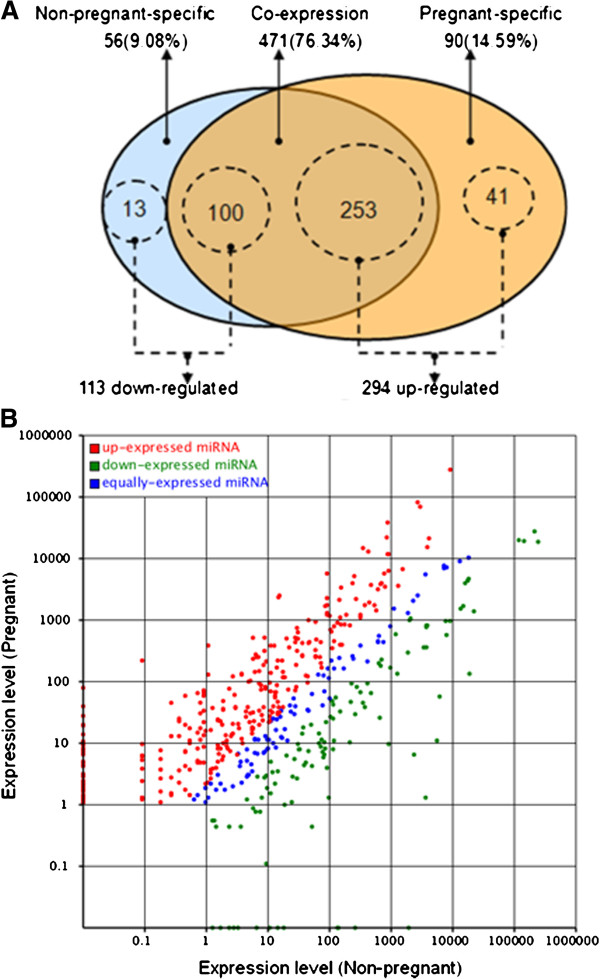
**Differentially expressed miRNAs in the non-pregnant and pregnant goat ovary libraries.** (**A**) Venn diagram displaying the distribution of 617 unique miRNAs in the non-pregnant (left, blue circle) and pregnant libraries (right, yellow circle). The overlapping region indicates co-expressed unique miRNAs; dashed circles indicate differentially expressed unique miRNAs in different libraries. (**B**) The scatter plot of differentially expressed miRNAs (control: X-axis, treatment: Y-axis). The X axis and Y axis show expression level of miRNAs in the two samples respectively. Red points represent miRNAs with ratio > 2; Blue points represent miRNAs with 1/2 < ratio ≤ 2; Green points represent miRNAs with ratio ≤ 1/2. Ratio = normalized expression of the treatment / normalized expression of the control.

As shown in Figure 3A, B and Additional file 6, 471 of 617 unique miRNAs (76.34%) were co-expressed in both libraries, and 90 (14.59%) and 56 (9.08%) of these miRNAs appeared to be preferentially expressed in the pregnant and non-pregnant libraries, respectively. Analysis of the sequencing data resulted in the identification of 407 unique miRNAs (out of 617, 65.96%) which were significantly differentially expressed in different libraries. Of these 407 unique miRNAs, 294 (41 pregnant-specific, 253 co-expressed) and 113 (13 non-pregnant-specific, 100 co-expressed) unique miRNAs were up-and downregulated in the pregnant library, respectively, compared to the non-pregnant library.

**Figure 4:**
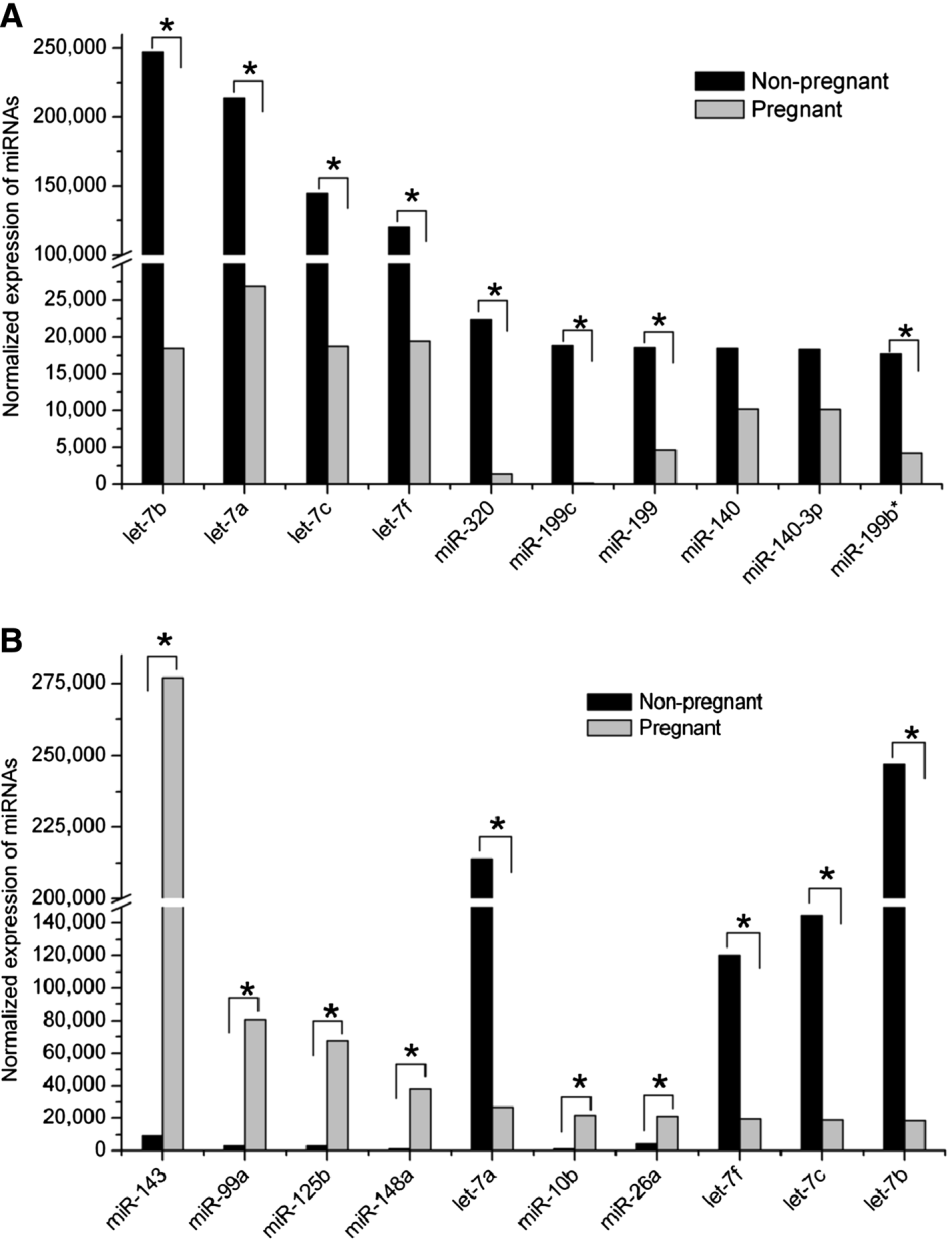
**Differential expression of the 20 most highly expressed miRNAs in the non-pregnant and pregnant goat ovary libraries.** (**A**) Differential expression of the 10 most highly expressed miRNAs in the non-pregnant library between both libraries. (**B**) Differential expression of the 10 most highly expressed miRNAs in the pregnant library between both libraries. * means a statistically significant difference (*P* < 0.05).

**Figure 5:**
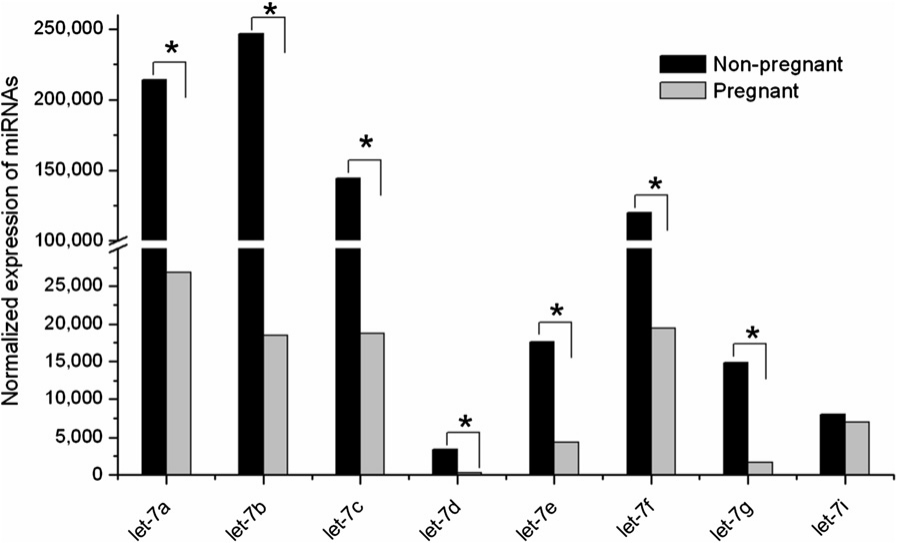
**Differential expression of the 8 members of let-7 family in the non-pregnant and pregnant goat ovary libraries.** * means a statistically significant difference (*P* < 0.05).

As shown in Figure 4A and B, the ten most highly expressed miRNAs in the non-pregnant library were all downregulated in the pregnant library, and of the ten most highly expressed miRNAs in the pregnant library, six miRNAs (miR-143, miR-99a, miR-125b, miR-148a, miR-10b, miR-26a) and four miRNAs (let-7a, let-7f, let-7c, let-7b) were down- and upregulated in the non-pregnant library, respectively. Moreover, eight members of the let-7 family (let-7a, let-7b, let-7c, let-7d, let-7e, let-7f, let-7 g and let-7i) were all identified in both libraries as show in Figure 5. These differential expression patterns of highly expressed miRNAs during pregnancy and non-pregnancy suggested that these miRNAs may be closely related to hircine ovarian development and function [17-20].

### MiRNA target prediction

**Figure 6:**
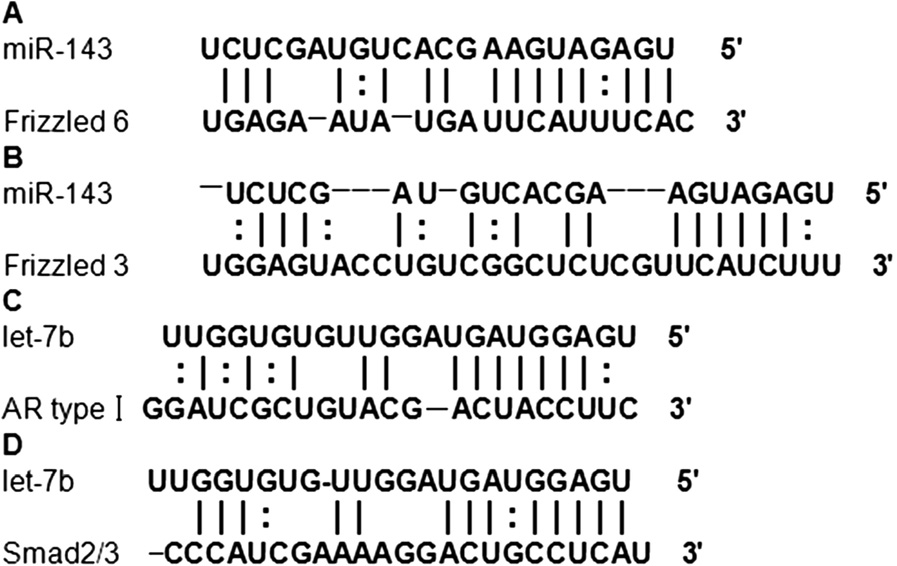
**Target prediction for selected miRNAs expressed in goat ovaries.** (**A**, **B**) Frizzled-6 and -3 were predicted as potential targets of miR-143. (**C**, **D**) Activin receptor I (AR type I) and Smad 2/3 were predicted as potential targets of let-7b.

MiR-143, the most highly expressed miRNA in the pregnant library (normalized expression level of 276,985), was predicted to bind the target sequences and repress expression of the Frizzled-6 and -3 receptor genes (Figure 6A and B), which may affect the classical Wnt/beta-catenin signaling pathway, indicating that miR-143 might play an important role in luteinization, progesterone secretion and the maintenance of pregnancy. In addition, let-7b, the most highly expressed miRNA in non-pregnant library (normalized expression level of 246,928), may target the Activin receptor I and Smad 2/3 genes in the TGF-beta signaling pathway (Figure 6C and D), and affect mammalian gonadal development, placentation and embryonic differentiation.

### Validation of hircine miRNAs

**Figure 7:**
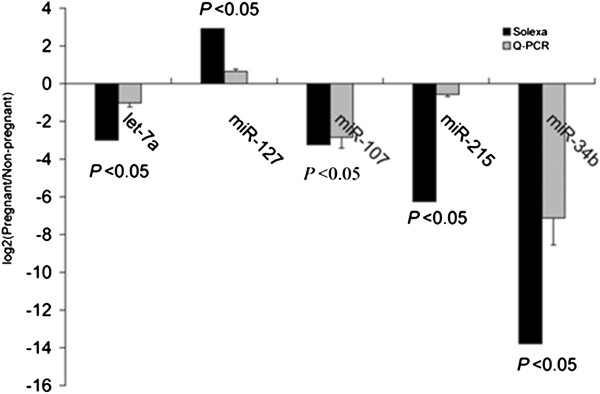
**Q-PCR validation of miRNAs identified in goat ovaries using Solexa sequencing technology.** Log_2_-ratio is the logarithm of the normalized miRNA expression (2^-∆Ct^) in the pregnant library divided by the normalized miRNA expression (2^-∆Ct^) in the non-pregnant library. Log_2_-ratio > 0 indicates upregulation in pregnant library compared to non-pregnant library; log_2_-ratio < 0 indicates downregulation in pregnant library compared to non-pregnant library. The *P* values are calculated based on a *t*-test of the replicate values (2^-∆Ct^) for each miRNA in the ovaries of pregnant and non-pregnant goats, and *P* < 0.05 indicates a statistically significant difference.

The expression levels of 5 randomly selected miRNAs were determined in the ovaries of pregnant and non-pregnant goats using q-PCR. MiR-127 was significantly upregulated in the pregnant hircine ovaries compared to the non-pregnant ovaries (*P* < 0.05). MiR-34b, miR-215, let-7a and miR-107 were all significantly downregulated in the pregnant hircine ovaries compared to the non-pregnant ovaries (*P* < 0.05). These expression patterns were consistent with the Solexa sequencing results (Figure 7 and Additional file 6).

## Discussion

Goats are one of the important economical animals, as they provide high-quality wool, meat and other products. However, hircine fecundity is relatively low, and this is a major constraint which prevents the development of the goat industry. Fecundity is a low heritability trait and conventional selection only supports slow improvement. Therefore, new methods to improve hircine fertility are highly desirable [21-23]. Molecular assisted breeding technology has been successfully used in the goat breeding industry, and the resource population of high fecundity of Anhui White goats was created successfully by our research group. In this study, we sequenced the small RNAs in the ovarian tissues of pregnant and non-pregnant Anhui White goats using Illumina Solexa technology to identify ovary-specific and differentially expressed miRNAs. Over eighty percent of known conserved mammalian miRNAs were found to be expressed in the ovaries of pregnant and non-pregnant goats (Figure 2A and C).

As the complete goat genome sequence has not yet been published, and goat-specific miRNAs are not recorded in the miRBase 18.0 database [24], we can currently only research hircine miRNAs using the miRNA sequences of closely related species (including mammals such as *Ovis aries*, *Bos taurus*, *Sus scrofa*, *Canis familiaris* and *Equus caballus*), or using goat EST sequences and Unigene information. Although 617 conserved miRNAs were found to be expressed in hircine ovaries using BLASTN in the study, the identification was highly dependent on the quantity and quality of the data in miRBase 18.0. Q-PCR was preformed to analyze the expression of 5 selected differentially expressed miRNAs in pregnant and non-pregnant hircine ovaries, and the results were consistent with the Solexa sequencing data. In theory, the expression levels of each candidate miRNA need to be validated using q-PCR; therefore, the 617 miRNAs identified in this study can only be regarded as a hircine ovary-specific miRNA reference dataset, and further research should be performed to validate the expression of miRNAs of interest in a larger number of samples. On the other hand, only one sample per group (pregnant or non-pregnant) was sequenced in this study, so no expression variances within the groups and intro-species could be estimated. To overcome this deficiency, we will add 1-2 samples in each physio-stage, or add samples with these conditions (before pregnancy, in pregnancy, after parturition) in the future, to make the library a complete process of pregnancy. Furthermore, we predicted 7 putative miRNAs using the goat EST database; however, the expression levels of these miRNAs in the libraries were very low. This discrepancy is due to the shortage of goat EST sequence information in the Genbank database, and is consistent with the previous studies [25,26]. With continual improvements in goat genome sequence information, we will be able to obtain richer, more accurate data on hircine miRNAs.

In the view of the different ovarian activity and endocrine function in pregnant and non-pregnant goats, differentially expressed miRNAs were identified in the ovaries of pregnant and non-pregnant goats which enabled to analyze the relationship between hircine miRNAs and reproductive traits such as follicular development, hormone secretion, luteinization and pregnancy maintenance. We identified 90 and 56 miRNAs which were specifically expressed in the ovaries of pregnant and non-pregnant goats, respectively. Some miRNAs which were not expressed in the ovaries of non-pregnant goats were expressed during pregnancy (Figure 3 and Additional file 6). MiRNAs usually regulate protein expression by binding to and repressing translation or promoting the degradation of their target mRNAs [27,28]. The present study indicated that upregulation of miRNAs in the ovaries of pregnant goats may inhibit the expression of target genes associated with follicular development, ovulation and estrogen secretion; thereby inhibiting ovulation and estrogen secretion. On the contrary, downregulation of other miRNAs in the ovaries of pregnant goats may relieve the repression of target genes associated with luteinization, progesterone secretion and pregnancy maintenance; thereby promoting luteinization and progesterone secretion.

Although much research has been performed on miRNAs in the gonads of pigs, cattle and other mammals, research of goat ovarian miRNAs has seldom been reported. In previous, miR-143 was found to be the most highly expressed miRNA in the testis and ovaries of Holstein cows, and 10 putative miRNA target genes involved in the GnRH-signaling and insulin-signaling pathways, which are associated with endocrine system function, were also identified [29]. Interestingly, in the study, miR-143 was also found to be the most highly expressed miRNA in the ovaries of pregnant goats (normalized expression level of 276,985). Target prediction indicated that miR-143 may bind to the Frizzled-6 and -3 receptor genes in the Wnt signaling pathway, thereby affecting the binding of Wnt-4 to its receptor (Figure 6A and B). Moreover, previous studies have suggested that the Wnt-4 gene, one of the most important members of the Wnt family, may regulate the function of ovarian granulosa cells and luteal cells by binding to specific members of the Frizzled receptor family [30,31]. Therefore, miR-143 may play an important role in reproduction, such as mammalian gonadal endocrine function and pregnancy maintenance. Other studies have also shown that miR-143 promotes normal adipocyte differentiation and fat deposition [2,32-34], indicating that miR-143 may play an important role in normal mammalian physical development, as well as mammalian reproduction.

It is a challenge to verify the complex functions of miRNAs. In the previous studies, miR-21 exerted an anti-apoptotic effect during the transformation of ovarian granulosa cells into luteal cells, and repression of miR-21 expression induced granulosa cell apoptosis and significantly reduced the rate of ovulation, via a mechanism dependent on luteinizing hormone secretion [35]. Also, miR-21 was demonstrated to express at significantly higher levels in the ovaries of Holstein cows compared to the testis (1.97-fold), indicating that miR-21 may play an important role in ovarian function [29]. Interestingly, the present study also showed that miR-21 was significantly upregulated in pregnant goat ovaries, compared to non-pregnant goat ovaries (1.92-fold). Increased LH secretion after becoming pregnant, followed by upregulation of miR-21 expression, may contribute to the transformation of ovarian granulosa cells into luteal cells. And the mechanisms regulating the anti-apoptotic effects of miR-21 in ovarian granulosa cells still need to be investigated further [36].

Recent research has indicated that specific members of the let-7 family can affect mammalian reproduction, development, cell proliferation and apoptosis [37-40]. In the present study, eight members of the let-7 family (let-7a, let-7b, let-7c, let-7d, let-7e, let-7f, let-7 g and let-7i) were expressed at high levels in both libraries, and let-7b was the most highly expressed miRNA in the ovaries of non-pregnant goats (normalized expression level of 246,928). Bioinformatics analysis indicated that let-7b may bind to the Activin receptor I and Smad2/3 genes in the TGF-beta signaling pathway, and may affect follicular development and estrogen secretion (Figure 6C and D).

## Conclusions

MiRNA expression patterns vary in the ovaries of pregnant and non-pregnant goats. In total, 617 conserved and 7 putative miRNAs were detected, and 407 differentially expressed miRNAs were identified in pregnant and non-pregnant ovaries, suggesting that miRNAs may play an important role in the regulation of goat ovarian function. For example, miRNA-143 was strongly associated with reproduction, and may potentially regulate the Wnt/beta-catenin signaling pathway by targeting Frizzled-6 and -3. Let-7b may regulate the TGF-beta signaling pathway by targeting the Activin receptor І and Smad2/3. Future work to characterize the expression of ovarian miRNAs at different stages of reproduction and in different breeds of goat, or in specific cell lines derived from ovarian tissues, is necessary to fully elucidate the functions of miRNAs in goat follicular development and hormone secretion, which will help to understand the relationships between miRNAs and mammalian reproduction, while enhancing the development of artificial reproduction and marker assisted selection (MAS) techniques in goats.

## Methods

### Ethics statement

Anhui White goats (a Chinese indigenous breed) were obtained from the College of Animal Science and Technology, Anhui Agricultural University, Hefei, China. Experiments were performed according to the Regulations for the Administration of Affairs Concerning Experimental Animals (Ministry of Science and Technology, China; revised in June 2004) and approved by the ethics committee of Anhui Agricultural University, Anhui, China, under permit No. AHAU20101025. The animals were allowed access to feed and water ad libitum under normal condition and were sacrificed humanely to minimize suffering.

### Ovary collection and total RNA isolation

The ovaries of Anhui White goats were collected and used to generate small RNA libraries. The collected ovaries were divided into two groups: six ovaries were from three 24-month old non-pregnant goats and six ovaries were from three 24 -month old pregnant goats. The ovaries were immersed in liquid nitrogen immediately after collection and stored at -80°C. Total RNA was isolated using TRIzol reagent (Invitrogen, Carlsbad, CA, USA), according to the manufacturer’s instructions. The quality of the total RNA was checked using the Agilent 2100 Bioanalyzer system (Santa Clara, CA, USA) and the samples were stored at -80°C until analysis.

### Small RNA library construction and sequencing

Two groups of total RNA were used for library preparation and sequencing by pooling equal quantity (10 μg) of total RNA isolated from six individual pregnant or non-pregnant goat ovaries. Briefly, total RNA were purified by polyacrylamide gel electrophoresis (PAGE) to enrich 15–35 nt molecules, then proprietary adapters were ligated to the 5^′^ and 3^′^termini of the RNAs and the samples were used as templates for cDNA synthesis. The cDNA was amplified using the appropriate number of PCR cycles to produce sequencing libraries, which were subsequently subjected to the proprietary Solexa sequencing-by-synthesis method using the Illumina Genome Analyzer (SanDiego, CA, USA). Sequencing was carried out at the Beijing Genomics Institute (BGI, Beijing, China).

### Data analysis

According to the principle of bioinformatics analysis, low-quality reads were removed from the raw reads. After trimming the 3^′^adaptor sequence, removing 5^′^ adaptor contaminants and counting the total, unique and length of reads, all valid sequences were obtained for further analysis. The overall flow of the sequencing data analysis is represented schematically in Additional file 4: Figure S3. All unique sequences were used to search the ncRNA data (Genbank, Repeat sequence and Rfam) with BLASTN to remove non-miRNA sequences (rRNA, tRNA, snoRNA, snRNA, etc.). Subsequently, the remaining sequences were analyzed using a BLAST search against miRBase 18.0. Sequences in the libraries with identical or related sequences (1~2 nucleotide substitutions permitted) to *Ovis aries* or other mammals (*Bos taurus*, *Sus scrofa*, *Canis familiaris*, *Equus caballus*) were identified as conserved miRNAs. Although the full goat genome sequence has not yet been published, we integrated data from the small RNA libraries with the goat EST sequences (http://www.ncbi.nlm.nih.gov/nucest) to identify goat putative miRNAs using the Mireap software (http://sourceforge.net/projects/mireap). Sequences with a perfect match or one mismatch were retained for further analysis. Subsequently, 60-80 nt of the EST sequences were extracted, and secondary structure was predicted and analyzed with Mireap using specific parameter settings [41].

To compare the differential expression of miRNAs in the ovaries of pregnant and non-pregnant goats, normalized expression (NE) of each miRNA was normalized to reads per million according to the total read count of the clean reads. When the normalized expression of a certain miRNA was zero between two samples, we revised its expression value to 0.01. If the normalized expression of a certain miRNA was lower than 1, further differential expression analysis was conducted without this miRNA. To compare the differential expression between the two samples, the fold-changes and *P*-values were used.

$$\mathrm{Fold}-\mathrm{change}=\log_{2}\left(\mathrm{Pregnant}-\mathrm{NE}/\mathrm{Non}-\mathrm{pregnant}-\mathrm{NE}\right).$$

P-value formula:

$$p\left(x|y\right)=\left(\frac{N_{2}}{N_{1}}\right){\frac{\left(x+y\right)!}{x!y!{\left(1+\frac{N_{2}}{N_{1}}\right)}^{\left(x+y+1\right)}}}_{D\left(y\geq y_{\max}|x\right)=\sum_{y\geq y_{\max}}^{\infty }p\left(y|x\right)}^{C\left(y\leq y_{\min}|x\right)=\sum_{y=0}^{y\leq y_{\min}}p\left(y|x\right)}$$

The x and y represent normalized expression level, and the N_1_ and N_2_ represent total count of clean reads of a given miRNA in small RNA library of ovaries of pregnant and non-pregnant goats, respectively [29]. Additionally, Hircine Unigene sequences (http://www.ncbi.nlm.nih.gov/unigene) were chosen to predict miRNA targets with RNAhybrid, using the parameter settings described by Rehmsmeier *et al*. (2004) [42].

### MiRNA validation via q-PCR

Quantitative PCR (q-PCR) was used to validate 5 randomly selected miRNAs that were differentially expressed by Solexa sequencing. One microgram of total RNA from each sample were reverse-transcript into cDNA using the miScript Reverse Transcription Kit (Qiagen, Dusseldorf, Germany) according to the manufacturer’s instructions. After incubation at 37°C for 1 h and deactivation at 95°C for 10 min, the mix was used as the template for q-PCR. Q-PCR was performed using standard protocols on the Roche LightCycler 480 II Real-Time PCR Detection System (Roche; LC480 II, Basel, Switzerland). In each assay, 1 μl of cDNA was added to 19 μl of mix containing 10 μl 2×SYBR green SuperReal PreMix (TIANGEN, Beijing, China), 0.4 μl of each primer and 8.2 μl RNase free H_2_O. The reaction was amplified at 95°C for 15 min, followed by 40 cycles of 95°C 10 s and 60°C 30 s. For each miRNA, three biological replicates were performed, and all of the reactions were run in triplicate. The cycle threshold was collected from each reaction, and the relative expression level of each miRNA to U6 snRNA was evaluated using the equation 2^-(CTmiRNA-CTU6RNA)^, and the fold-change (log_2_-ratio) and *P* value were used to show the differential expression of miRNA in the two samples [43-45]. The miRNA-specific primers were presented in Additional file 7.

## Competing interests

The authors declare that they have no competing interests.

## Authors’ contributions

These studies were designed by XDZ, YHZ, JPD and XRZ. XDZ carried out all the experimental analyses and prepared all figures and tables. XDZ and YHZ analyzed the data and drafted the manuscript. YHL, YL, HGC and ZJY contributed to revisions of the manuscript. JPD and XRZ assisted in explaining the results and revised the final version of the manuscript. All authors have read and approved the final manuscript.

## Supplementary Material

Additional file 1 Distribution of counts of the sequencing results originating from known RNA classes in non-pregnant and pregnant libraries. Click here for file

Additional file 2 Conserved miRNAs and miRNA*s in the ovaries of non-pregnant goats. The miRNA: miRNA* duplexes were marked in red font. Click here for file

Additional file 3 Conserved miRNAs and miRNA*s in the ovaries of pregnant goats. The miRNA: miRNA* duplexes were marked in red font. Click here for file

Additional file 4: Figure S1 The length distribution of unannotated sequences in non-pregnant and pregnant libraries. **Figure S2.** The stem loop structures of precursors of predicted miRNA candidates. The red line parts indicate the mature sequences. (A) Non-pregnant; (B) Pregnant. **Figure S3.** Analysis workflow of the Solexa sequencing results. Click here for file

Additional file 5 Identification of goat putative miRNAs from goat EST sequences. Click here for file

Additional file 6 Differential expression of conserved miRNAs and miRNA*s in the ovaries of non-pregnant and pregnant goats. (1). -std. represents normalized expression level of miRNA in a sample. Normalized expression = Actual miRNA count/Total count of clean reads*1,000,000. (2). Sig-label: ** represents fold change (log_2_) > 1 or fold change (log_2_) < -1, and *P*-value < 0.01; * represents fold change (log_2_) > 1 or fold change (log_2_) < -1, and 0.01 ≤ *P* < 0.05; None represents others. Fold change = log_2_ (Pregnant std. / Non-pregnant std.) (3). miRNAs in red font used for the q-PCR analysis. Click here for file

Additional file 7 Primer sequences for q-PCR experiments. Click here for file
