# Supplementary material for: Characterization and differential expression of microRNAs in the ovaries of pregnant and non-pregnant goats (Capra hircus)
Source: BMC Genomics. 2013 Mar 7;14:157. doi: 10.1186/1471-2164-14-157 (PMC3599660; doi:10.1186/1471-2164-14-157)
Supplement: Additional file 4: Figure S1 — The length distribution of unannotated sequences in non-pregnant and pregnant libraries. Figure S2. The stem loop structures of precursors of predicted miRNA candidates. The red line parts indicate the mature sequences. (A) Non-pregnant; (B) Pregnant. Figure S3. Analysis workflow of the Solexa sequencing results. [file 1471-2164-14-157-S4.pdf]

Additional file 4.

Figure S1. The length distribution of unannotated sequences in non-pregnant and pregnant libraries

Figure S2. The stem loop structures of precursors of predicted miRNA candidates.
The red line parts indicate the mature sequences. (A)Non-pregnant (B) Pregnant

Figure S3. Analysis workflow of the Solexa sequencing results

Figure S1

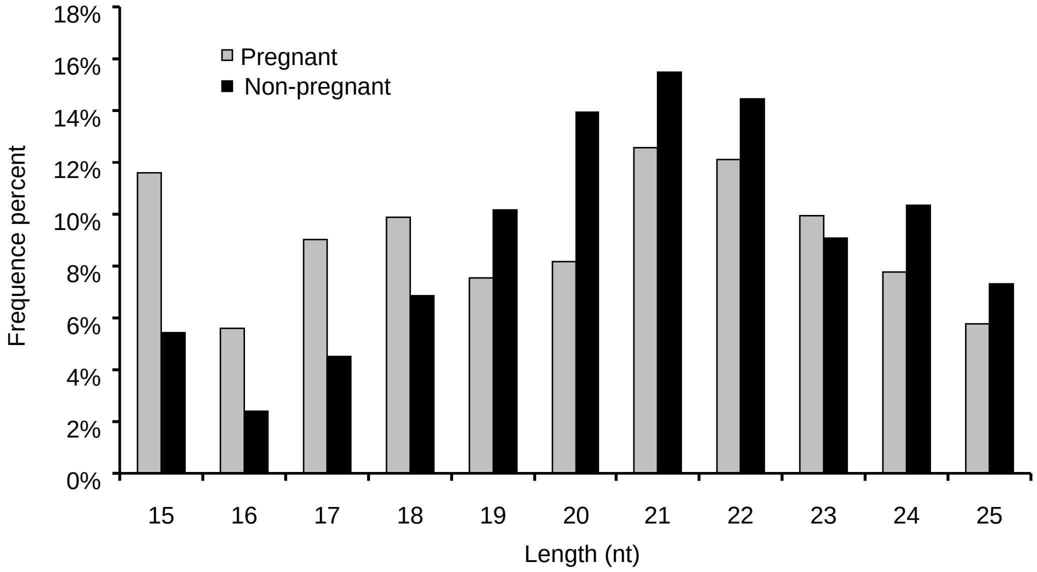

Figure S2

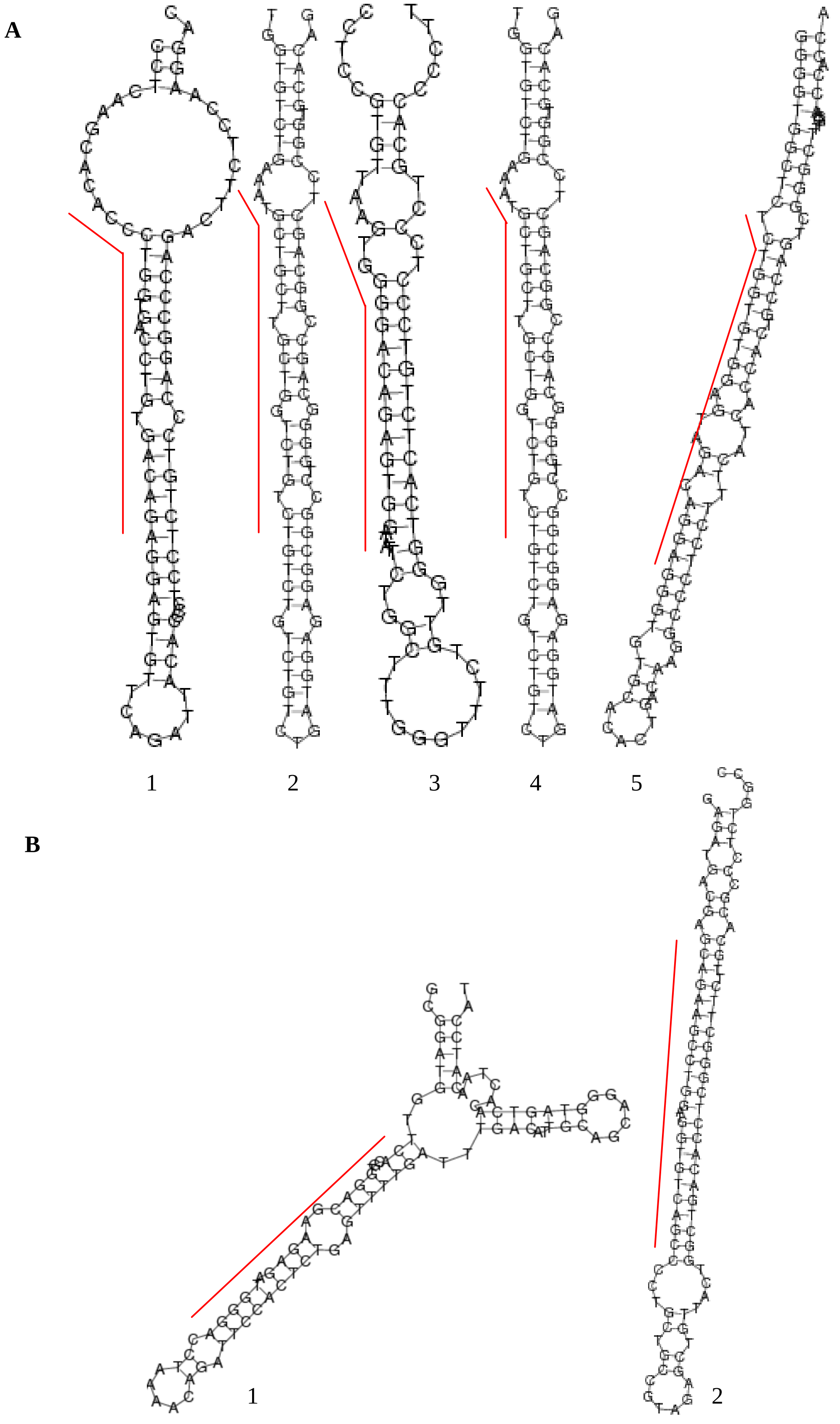

Figure S3

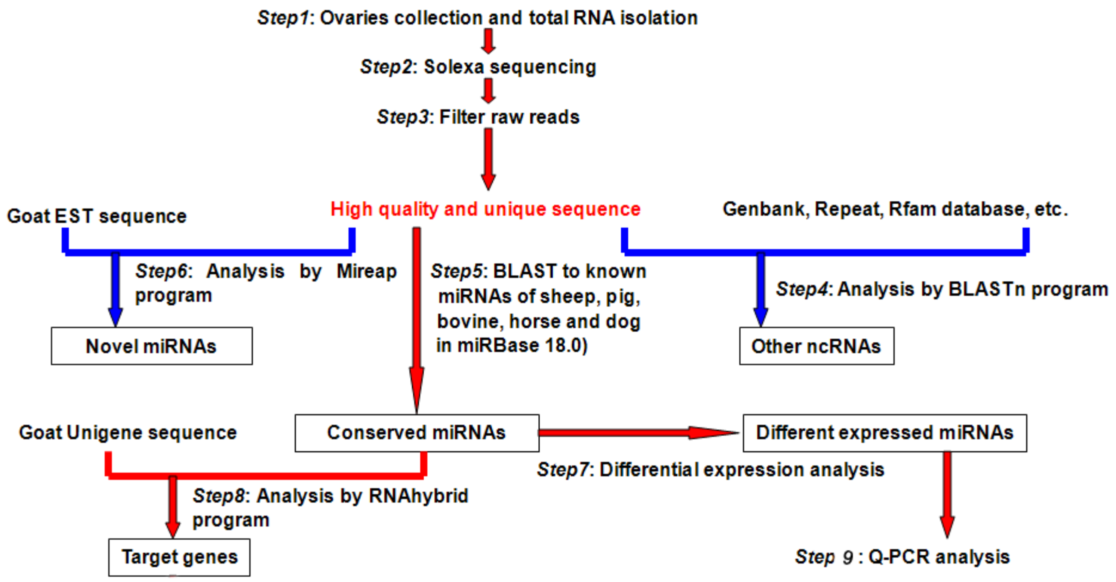

Step1: Ovaries collection and total RNA isolation
Step2: Solexa sequencing
Step3: Filter raw reads
Goat EST sequence
High quality and unique sequence
Genbank, Repeat, Rfam database, etc.
Step6: Analysis by Mireap program
Step5: BLAST to known miRNAs of sheep, pig, bovine, horse and dog in miRBase 18.0)
Step4: Analysis by BLASTn program
Novel miRNAs
Other ncRNAs
Goat Unigene sequence
Conserved miRNAs
Different expressed miRNAs
Step7: Differential expression analysis
Step8: Analysis by RNAhybrid program
Target genes
Step 9 : Q-PCR analysis
